# Myocardial Bridging in Sports and Exercise Cardiology: Screening Challenges, Clinical Management, and Physical Activity Guidance

**DOI:** 10.3390/jcm15176580

**Published:** 2026-08-26

**Authors:** Jonathan C. Spaan, Sean P. Swearingen

**Affiliations:** Cardiology, Rush University Medical Center, Chicago, IL 60612, USA; jonathan_c_spaan@rush.edu

**Keywords:** myocardial bridging, sports cardiology, sudden cardiac death, athlete screening, coronary anomaly, exercise-induced ischemia, physical activity guidance, left anterior descending artery, intravascular ultrasound, coronary computed tomography angiography

## Abstract

Myocardial bridging (MB) is a congenital coronary anomaly in which a segment of an epicardial coronary artery courses intramurally beneath the myocardium for part of its length, resulting in dynamic compression during systole. Once considered a largely benign incidental finding, accumulating evidence has demonstrated MB to be a clinically significant substrate for myocardial ischemia, arrhythmia, and sudden cardiac death (SCD), particularly under conditions of elevated adrenergic drive such as vigorous physical exercise. The prevalence of MB is highly dependent on the diagnostic modality used, ranging from approximately 0.5–2.5% on coronary angiography to 15–85% on post-mortem and advanced intravascular imaging studies, reflecting a substantial detection gap- in particular on routine cardiac screening tests. This review critically examines the current evidence base regarding MB in the context of competitive and recreational sport. We address: (1) the pathophysiology of exercise-induced hemodynamic compromise in MB; (2) the limitations of existing screening modalities—including electrocardiography, echocardiography, exercise stress testing, and advanced coronary imaging in identifying athletes at risk; (3) the clinical management of symptomatic MB, encompassing pharmacological therapy, coronary revascularization strategies, and catheter-based interventions; and (4) physical activity guidance for athletes with known or suspected MB.

## 1. Introduction

The clinical significance of MB has been debated for decades. Early autopsy studies by Reyman in 1737 first described the anatomical variant, but its potential for dynamic compression was not discovered until much later [1]. In modern cardiology, MB gained renewed attention following reports of MB-related ischemia, ventricular tachycardia, and sudden death—including in young, otherwise healthy athletes [2,3]. Post-mortem series from programs screening for SCD have identified MB as a contributing or primary cause in a subset of young deaths, particularly in the context of exercise [4,5].

Despite this, MB remains poorly integrated into mainstream sports cardiology practice. The condition is frequently dismissed as an incidental finding when detected in asymptomatic athletes, and there is no international consensus on screening, risk stratification, or return-to-play decision-making. The 2020 ESC Guidelines on Sports Cardiology and Exercise in Patients with Cardiovascular Disease make limited reference to coronary anomalies generally and provide very broad MB-specific recommendations [6]. The latest statement from the American Heart Association/American College of Cardiology guidance on eligibility for athletes with cardiovascular abnormalities allows for return to play with no specific recommendations for further testing or risk/benefit discussion when bridging is discovered in an asymptomatic athlete [7].

This review aims to synthesize the current evidence on MB in the sports cardiology setting, critically evaluate the options for diagnosis and possible treatment, as well as highlight the knowledge gaps that must be addressed to protect athlete welfare. We present an evidence-based framework for physical activity guidance while acknowledging the limitations imposed by the current paucity of robust longitudinal data. The evidence for this study was obtained through assessing major literature and reviews between 1980 and 2025. The literature was primarily discovered through first assessing review articles in major cardiology journals such as the journal of the American College of Cardiology and European Heart Journal and their associated references. Decision to include articles were based on study quality and relevance to the arguments presented in the paper.

## 2. Pathophysiology of Myocardial Bridging and Exercise

### Anatomical and Hemodynamic Basis

In individuals with MB, the intramural arterial segment is subject to extrinsic compression during ventricular systole. Under resting conditions, this compression may be hemodynamically insignificant because coronary flow is predominantly diastolic. However, the degree of functional compromise is influenced by several factors: bridge length (typically 10–30 mm but ranging up to 80 mm), depth of the tunneled segment within the myocardium, the degree of systolic compression on angiography or fractional flow reserve (FFR) assessment, and the presence of concomitant atherosclerosis [1,8,9].

The hemodynamic impact of MB is substantially amplified during exercise. Exercise-induced tachycardia shortens diastolic filling time, compressing the window during which the bridged segment is patent. Simultaneously, sympathetically mediated increases in myocardial contractility deepen and prolong systolic compression of the tunneled vessel. Studies using intracoronary pressure wire assessment have demonstrated that FFR across a bridged segment can deteriorate significantly from rest to pharmacologically simulated exercise conditions, crossing the ischemic threshold of <0.80 in a meaningful proportion of patients who appear non-obstructive at rest [10]. A major contributing factor of this hemodynamic compromise is the fact that systolic compression will frequently extend into early diastole due to delayed relaxation of the overlying muscle, which is exacerbated during activity [11].

Further compounding this obstruction, endothelial dysfunction has been documented within and proximal to MB segments. The abnormal shear stress environment within the tunnel predisposes to local vasospasm and may accelerate atherosclerosis immediately proximal to the bridge—a paradox in which the bridged segment itself is relatively protected from plaque formation but the proximal vessel is at heightened atherosclerotic risk [9,12]. This proximal plaque formation can further compromise coronary perfusion.

**Figure 1:**
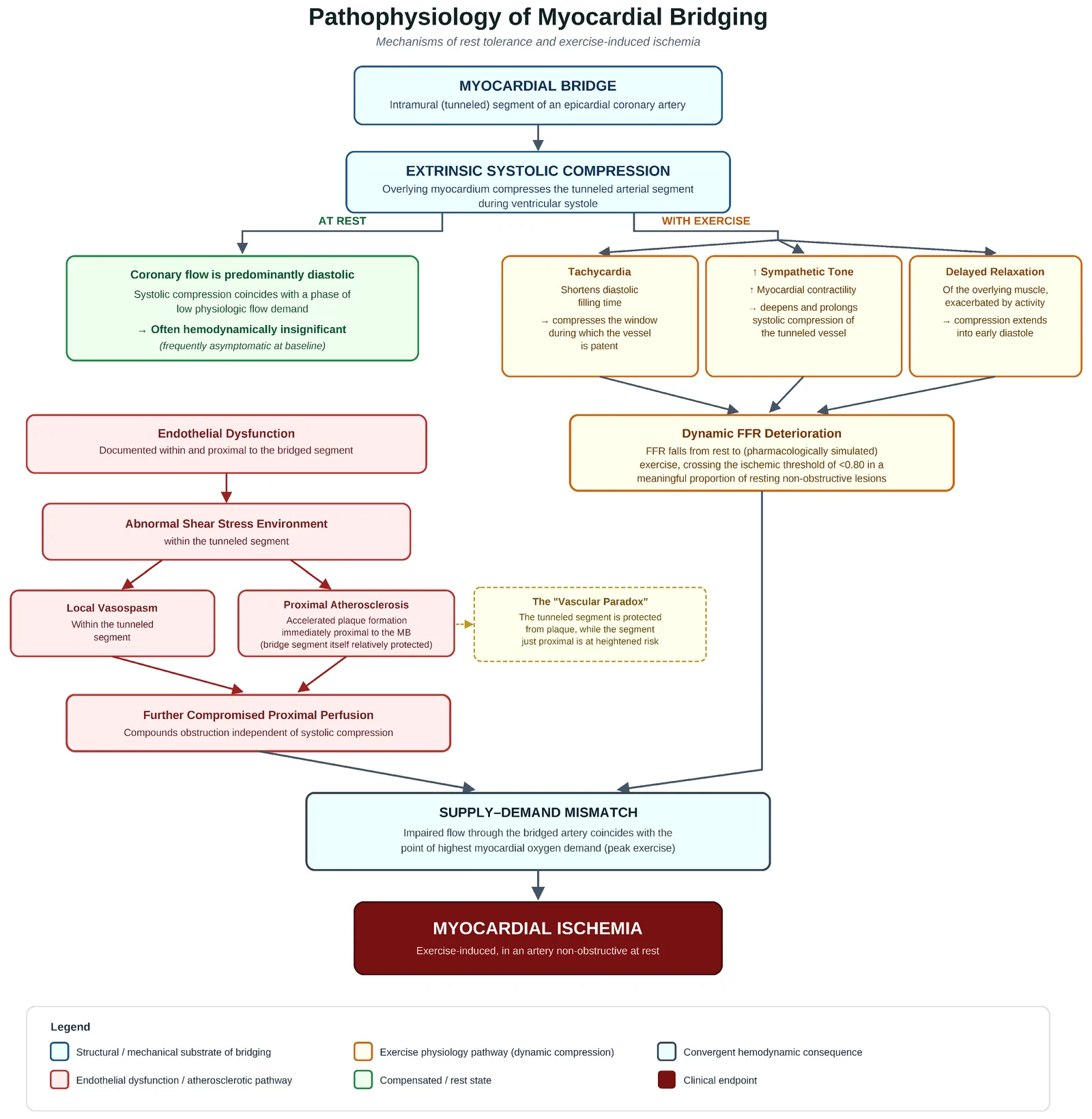
Pathophysiology of myocardial bridging. Although this phenomenon is clinically silent at rest, the physiologic changes with exercise combined with the endothelial dysfunction proximal to the bridge results in supply–demand mismatch and eventual myocardial ischemia [1,8,9,10,11,12].

Ultimately, this impaired blood flow through a coronary artery during exercise due to myocardial bridging occurs during a time when the myocardium is at its highest demand for oxygen. This results in a supply–demand mismatch for the myocardial tissue resulting in myocardial ischemia [1]. The detailed process through which this occurs is shown in Figure 1.

## 3. Epidemiology and Prevalence in Athletic Populations

The true prevalence of MB in the general population and in athletes specifically remains poorly defined, largely because prevalence estimates vary by at least two orders of magnitude depending on the detection modality employed. Coronary angiography detects MB in approximately 0.5–2.5% of patients, a figure that substantially underestimates anatomical prevalence due to its reliance on hemodynamic compression to produce a visible ‘milking effect’ [1]. Autopsy series report a wide variety in prevalence ranging from 15 to 85% [13,14,15]. Coronary computed tomography angiography (CCTA) and intravascular ultrasound (IVUS) occupy intermediate positions, with CCTA studies reporting the prevalence of 3.5–30% in symptomatic populations [16,17].

The frequency of myocardial bridging as a cause for SCD in athletes remains difficult to clearly establish as well; however, several studies clearly show that a myocardial bridging is present in a significant number of athletes following SCD. SCD registries in competitive athletes have implicated MB in approximately 1–4% of exercise-related SCD cases where structural pathology is identified [18,19,20].

A critical epidemiological gap is the absence of prospective prevalence data in healthy athletic cohorts using advanced imaging. We currently cannot determine what proportion of competitive athletes harbor hemodynamically significant MB, nor can we identify the exact proportion of these that will experience adverse events.

## 4. Screening Challenges in Sports Cardiology

### 4.1. Standard Pre-Participation Screening

Pre-participation cardiovascular screening programs for athletes—whether they incorporate just a history and physical or also include an EKG—are not designed to detect MB. The condition is clinically silent in the majority of affected individuals and typically leaves no specific ECG abnormalities [1].

The 2017 international criteria improved the specificity of ECG interpretation in athletes, but even with these guidelines, EKG abnormalities from myocardial bridging remain difficult to detect. Given that MB typically only causes cardiovascular affects during activity, it is not common to see changes related to MB on a resting EKG. Additionally, athlete-specific EKG criteria may decrease the specificity for identification in the rare circumstances where baseline EKG abnormalities are present. Anterior T-wave inversion, which might represent MB-related ischemia, may overlap with normal repolarization patterns in trained athletes [21]. This diagnostic ambiguity could provide false reassurance in an athlete who has MB.

### 4.2. Exercise Stress Testing

Exercise stress testing (EST) represents an intuitively appealing screening tool for MB given the exercise-dependent nature of the pathophysiology of this disease. However, the sensitivity of conventional EST for MB-related ischemia is modest. False-negative results are common because the degree of compression required to produce visible ST segment changes on standard stress ECG or stress myocardial perfusion imaging may not be achieved during a standardized exercise protocol [22,23]. This problem is amplified in well-conditioned athletes who tolerate high workloads and are likely not being pushed as hard as is necessary on a standard stress test. It is worth noting, however, that individuals with longer and deeper myocardial bridges are more likely to have ST segment EKG changes [23]. Additionally, there is data to show that MB patients are also more likely to have other ischemic findings on their EKG such as an increase in ventricular ectopy and prolonged QTc [24].

Exercise stress echocardiography offers further sensitivity compared to stress EKG and is a test that can easily be performed in young athletes given the absence of any radiation exposure. However, the temporal resolution of echocardiography during peak exercise is limited, and subtle regional dysfunction may be missed. Ultimately, the sensitivity of stress echo is low, with recent data from a group of middle aged symptomatic patients showing a sensitivity of less than 20% identifying abnormalities in patients with a myocardial bridge [25].

### 4.3. Advanced Coronary Imaging

Coronary computed tomography angiography (CCTA) has emerged as the most reliable non-invasive modality for anatomical MB detection, capable of defining bridge length, depth, and the degree of myocardial coverage [16,17]. The spatial resolution of modern CCTA (0.6 mm isotropic voxels on third-generation dual-source scanners) allows reliable identification of bridges ≥10 mm in length. However, CCTA provides anatomical rather than functional information; the presence of a MB on CCTA does not establish hemodynamic significance as systolic compression cannot be assessed on standard coronary CTA. Radiation exposure, though substantially reduced with newer acquisition protocols (as low as 1–3 mSv with prospective ECG gating), remains a concern for population-level screening in young athletes.

Invasive coronary assessment with intracoronary pressure wire (FFR measurement) and IVUS or optical coherence tomography (OCT) provides the most comprehensive characterization of MB hemodynamics. FFR across the bridged segment identifies functionally significant bridges with greater precision than angiography alone [26]. Dobutamine provocation, which more closely mimics exercise physiology, may unmask hemodynamically significant bridges not apparent on standard FFR protocols [10]. These are invasive procedures that carry procedural risk, and their use in asymptomatic or mildly symptomatic athletes is ethically and practically challenging. IVUS directly visualizes the intramural course and peri-bridge atherosclerosis, adding important anatomical detail, but again requires invasive access [26].

### 4.4. The ‘Detection Gap’ and Its Clinical Consequences

The fundamental problem confronting sports cardiology screening is the vast ‘detection gap’ between the anatomical prevalence of MB and its clinical identification. The majority of athletes with MB will never be detected unless they present with symptoms or an adverse event. Of those detected incidentally—for example, during CCTA performed for chest pain evaluation—risk stratification tools are absent. There is no validated scoring system, clinical algorithm, or biomarker panel that can reliably identify which detected MB represents a clinically significant substrate in an athlete. This creates an immediate clinical dilemma: should an incidentally detected MB alter participation recommendations, and if so, by how much?

The answer is currently empirical at best. The absence of prospective cohort data means that decisions are largely based on expert opinion, case series, and extrapolation from non-athletic populations. A systematic review by Ural et al. identified only observational data of individuals with known myocardial bridging, with no randomized trial evidence available [27]. This evidence vacuum means that conservative and liberal approaches to athletic participation both lack an evidence base, and that athletes and their physicians are making decisions under conditions of genuine uncertainty.

## 5. Clinical Management of Myocardial Bridging

### 5.1. Pharmacological Therapy

The pharmacological management of symptomatic MB centers on reducing heart rate and enhancing diastolic coronary filling time, thereby mitigating the exercise-related hemodynamic compromise described above. Beta-blockers represent first-line therapy and have demonstrated symptomatic benefit in observational studies [1,8]. By reducing resting and exercise heart rate, beta-blockers prolong diastole, increase the proportion of the cardiac cycle during which the bridged segment is patent, and attenuate sympathetically driven increases in contractility that worsen systolic compression. Non-selective beta-blockers (e.g., propranolol, nadolol) may offer advantages over cardioselective agents in this context, though head-to-head data are absent.

Non-dihydropyridine calcium channel blockers (verapamil, diltiazem) represent a second-line option, exerting negative chronotropic effects similar to beta-blockers while additionally reducing coronary vasospasm, which may co-exist with MB. Case series support symptomatic efficacy, but no comparative trial has evaluated beta-blocker versus calcium channel blocker therapy in MB. Importantly, both drug classes have exercise-limiting effects that must be considered in competitive athletes for whom performance capacity is central to participation decisions.

Unfortunately, for the very reason these medications are effective, they tend to be poorly tolerated in the athletic population. Although improved diastolic filling is beneficial to athletes, minimizing their maximum heart rate diminishes their maximum cardiac output as well as their VO2 max—a phenomenon that has been well studied for years [28]. A drop in cardiac output and VO2 max will lead to a drop in performance from an athlete which is something that can be extremely frustrating even to recreational athletes.

Despite the challenges of over-the-counter medical therapy, there is a more conservative therapeutic intervention that may both minimize symptoms from myocardial bridging while also improving exercise performance: aggressive hydration. In situations where hydration is poor, this decreases coronary perfusion pressure and lead to easier compression of a coronary artery from a myocardial bridge. There is limited data in prior case reports describing athletes with MB who present with symptoms during prolonged exertion (rather than specifically a high heart rate), which resolved with aggressive IV fluids [29,30]. Ensuring proper hydration before, during and after all workouts can potentially allow physicians to avoid the challenging risk/benefit decisions of initiating medications for myocardial bridging.

### 5.2. Coronary Revascularization

Given the inability of athletes to tolerate medical therapy for myocardial bridging, procedural intervention becomes a possible attractive option for those who are symptomatic. Surgical myotomy—division of the myocardial fibers overlying the bridged segment—has been performed in symptomatic patients refractory to medical therapy since the 1970s, with several case series reporting relief of ischemia and symptom resolution [31,32]. In experienced surgical centers, operative mortality is low and mid-term outcomes have been favorable [33]. However, surgical myotomy carries inherent procedural risk including coronary injury, bleeding, and requirement for cardiopulmonary bypass in some cases. The evidence base consists entirely of retrospective case series; no prospective or randomized data exist. Patient selection criteria are heterogeneous across reported series, limiting generalizability.

Coronary artery bypass grafting (CABG) to the mid-LAD beyond the bridge has also been employed in situations where atherosclerotic obstruction is present in addition to a myocardial bridge. However, in cases of isolated myocardial bridging alone, competitive flow from the native vessel through the compressed bridge may limit graft patency, and given the success of myotomy in these cases, this is the preferred option [33].

Percutaneous coronary intervention (PCI) with stenting of the bridged segment has been attempted but is associated with poor outcomes, including high rates of in-stent restenosis, stent fracture, and coronary perforation—consequences of the repeated mechanical compression imposed on an implanted scaffold by each ventricular systole [34]. PCI is not recommended for MB and should be avoided except in exceptional circumstances.

### 5.3. Risk Stratification and Challenges with the Athletic Population

In symptomatic patients, management decisions for MB are guided by the severity of ischemia on functional testing, the degree of angiographic compression, FFR values, the presence of proximal atherosclerosis, and response to medical therapy. The athletic population, however, poses additional challenges. To begin with, athletes are regularly exposing themselves to higher than normal cardiovascular demands during their training, and typically their hearts have undergone significant physiologic changes after prolonged time playing a particular sport. This combination of factors places them in a situation where they are less likely to experience symptoms at lower workloads, but at an overall higher risk for sudden cardiac death due to how regularly they are maximally exerting themselves.

Intuitively, if an athlete has MB and is significantly symptomatic, it makes sense to treat them with either medications if tolerated, or a possible surgery as discussed previously depending on the risk/benefit discussion on how important it is for that athlete to return to activity. The true challenge lies in management of athletes when a myocardial bridge is discovered, and an athlete has either questionable symptoms or no symptoms at all.

Given the relatively high frequency of myocardial bridging, it is important to identify specific risk factors that may make one myocardial bridge more dangerous to an athlete than another. There is currently no validated risk stratification score for MB. Proposed risk factors for adverse events—including bridge length >25 mm, myocardial depth >2 mm, systolic FFR <0.76, proximal atherosclerosis, and evidence of ischemia on functional testing—have been derived from case series and small cohort studies and have not been prospectively validated [1,10,26]. The independent and combined prognostic value of these parameters in athletic populations is unknown, but they are factors that should get taken into final decision-making on management with athletes as is discussed below.

## 6. Physical Activity Guidance for Athletes with Myocardial Bridging

### 6.1. The Evidence Vacuum

Physical activity guidance represents a clinically pressing and simultaneously evidence-deficient domain in MB management for athletes. The fundamental challenge is between the established cardiovascular benefits of regular physical activity and exercise—including competitive sport—and the theoretical risk of exercise-induced ischemia, arrhythmia, and SCD in athletes harboring hemodynamically significant MB. Currently, no prospective study has evaluated athletic outcomes in cohorts with confirmed MB stratified by participation level, no randomized trial has compared exercise restriction versus unrestricted participation, and no natural history data exist to quantify the absolute risk of adverse events per year of athletic participation in MB-positive athletes. In this evidence vacuum, guidance has been issued on the basis of case reports, expert consensus, and general principles of exercise physiology. The consequence is substantial heterogeneity in recommendations between clinicians.

### 6.2. Current Guideline Positions

The 2020 ESC Sports Cardiology Guidelines addresses myocardial bridging in athletes with a recommendation to obtain a maximum effort exercise stress test to assess for ischemia in asymptomatic athletes where a myocardial bridge is discovered [6]. There is Class IIa (Level C) evidence to encourage both continued leisure and competitive activities if that testing is normal. In the case of abnormal testing (either from symptoms, ST changes or arrhythmias) it is recommended that patients do not compete, and recreational exercise can be considered on a case-by-case basis. Competitive activity may be considered once proper revascularization has occurred. The 2025 AHA/ACC Guidelines for Clinical Consideration for Competitive Sports Participation for Athletes with Cardiovascular Abnormalities has an even more lenient recommendation, suggesting that in an asymptomatic athlete with myocardial bridging, no further cardiovascular testing is recommended [7].

### 6.3. Proposed Framework for Physical Activity Decision-Making

Although the overall frequency of myocardial bridging being implicated in sudden cardiac death due to activity is low, it is clear that it does occur—even in individuals who were previously asymptomatic. As a result, it seems reasonable to have a screening and monitoring program when bridging is discovered in asymptomatic individuals. By drawing on physiological principles, as well as available and observational data presented above, we present a frame work for how to manage these individuals. This framework is provisional and should be superseded by prospective data as it emerges.

Step 1—Establish Hemodynamic Significance:

Not all anatomically detected MB is functionally significant. Athletes with incidentally detected MB on CCTA should undergo functional evaluation to determine hemodynamic relevance. This should include maximal exercise stress testing with imaging (stress echocardiography imaging preferred over ECG-only exercise stress testing), and aggressive follow-up assessment of symptoms during sport-specific activity from the patient’s trainer and coaching staff with regular sports cardiology outpatient follow-up.

Step 2—Characterize Additional Risk Factors:

In the case of a normal stress test and bridge characteristics (length, depth, angiographic compression grade), proximal atherosclerosis, family history of premature coronary disease or unexplained SCD, and the athlete’s competitive level and sport type (endurance vs. high-intensity intermittent vs. static) should all be integrated into the risk assessment. Endurance sports involving sustained high heart rates (long-distance running, cycling, triathlon) may impose greater sustained coronary hemodynamic demand than intermittent high-intensity sports, though evidence for this differential risk is lacking.

Step 3—Stratify and Advise:

It would be reasonable for athletes with no demonstrable ischemia, no arrhythmia, no proximal atherosclerosis, and low-risk bridge characteristics (short, shallow) to participate in sport with medical follow-up and education regarding warning symptoms. Athletes with evidence of ST changes or mild ventricular ectopy should receive optimized medical therapy with beta-blocker or calcium channel blocker and undergo reassessment of ischemic burden on treatment before a return-to-play discussion occurs. If an athlete is able to tolerate this medical therapy and still able to compete at their desired level with completely normal follow-up stress testing, an extensive shared decision-making discussion should occur between the physician and the athlete in regard to further competition as is discussed below.

In cases of severe ST changes or sustained ventricular arrhythmias on stress testing, strong consideration should be made for a surgical myotomy prior to return to competitive sports even if the athlete is asymptomatic when this occurs. This option should especially be considered for athletes with higher risk bridge characteristics. Typically, the longer and deeper the bridge the higher the risk, but based on prior data, typically a length greater than 25 mm or a depth greater than 2 mm [26] would be considered reasonable cutoffs for “higher risk” findings.

Step 4—Shared Decision-Making and Ongoing Monitoring:

A shared decision-making process—in which the athlete is fully informed of the uncertainties, the theoretical risks, and the potential benefits and harms of restriction versus participation—is an essential component of any sports cardiology discussion. Athletes should be equipped with individualized action plans, trained support staff should be aware of the diagnosis, and AED accessibility at training and competition venues must be confirmed. Annual reassessment, including repeat functional testing and ECG review, should be standard for all athletes even with incidentally discovered myocardial bridging and normal stress testing.

For those athletes with either positive stress testing that is resolved with medications or who are asymptomatic with normal stress testing but high risk bridge characteristics routine monitoring with heart rhythm monitors should be performed on an annual basis during competition and strong consideration should be made for athletes to undergo repeat annual stress EKGs on a yearly basis.

Additional Considerations—The Symptomatic Athlete:

Athletes with severe or refractory ischemia on stress testing, prior syncope during exercise, or resuscitated cardiac arrest require consideration of surgical myotomy before return to competitive sport. Additionally, for those athletes in whom medical therapy is being attempted but they are not able to tolerate it, strong consideration for surgical myotomy should be considered as well. A multidisciplinary discussion including sports cardiologists and cardiac surgeons is essential to this process.

**Figure 2:**
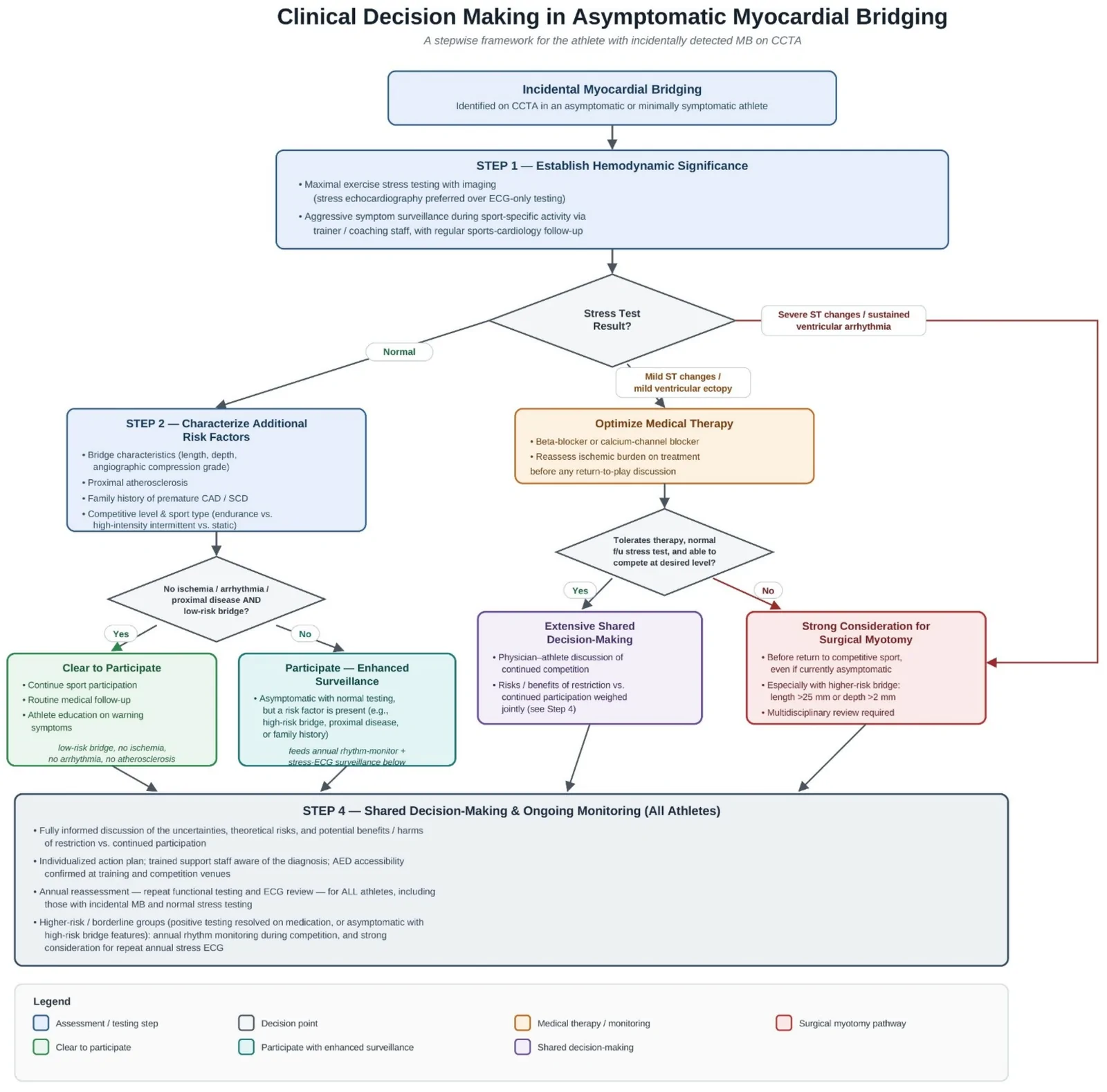
Clinical decision-making in asymptomatic myocardial bridging. For athletes with this condition, obtaining a routine stress test can allow for improved risk stratification for possible need for medical or procedural interventions as well as return-to-play decisions.

Invasive FFR assessment with dobutamine provocation could be considered in athletes with significant symptoms or in elite competitors where precision of risk assessment is paramount and where the consequences of unnecessary restriction are substantial. Given the invasive nature of this procedure, it would require and extensive risk/benefit discussion. A stepwise process for this decision-making pathway is shown in Figure 2.

## 7. Discussion

This review summarizes the pathology and management of a common anatomical cardiovascular variant that is still of unclear clinical significance and is not easily diagnosed on routine screening tests—a combination that poses particular challenges in sports cardiology.

Three overarching problems emerge from this evidence synthesis. First, there is no reliable method of screening for clinically significant MB in an athletic population. Standard pre-participation programs will not detect MB, and even if they did, no validated tool exists to distinguish the athlete who will experience an adverse event from the one who will complete a career of competitive sport without incident. Second, the therapeutic landscape for MB—while offering several pharmacological and procedural options—rests on an evidence base that would be considered inadequate for almost any other cardiovascular condition. The management of symptomatic MB in athletes is guided by case reports and expert opinion, not randomized trial data. Third, physical activity guidance is the clinical question most frequently asked by athletes and physicians following an MB diagnosis, and it is the question for which evidence is most completely absent.

The relative rarity of SCD events that can be specifically attributed to MB, paradoxically, makes this problem harder to solve. Because the absolute event rate is low, extremely large prospective cohorts would be required to generate meaningful outcome data in a reasonable timeframe. This has the practical consequence that individual institutions cannot accumulate sufficient numbers, and international collaborative registries are essential. The infrastructure for such registries exists in European sports cardiology networks (the European Association of Preventive Cardiology, the Sports Cardiology section of the ESC), but MB has not yet been prioritized as a registry target condition.

In the absence of large data sets to help determine the exact risk of cardiovascular complications due to myocardial bridge, clinicians are left with the challenge of appropriately utilizing medical resources when this is discovered incidentally. The frequency of these incidental discoveries will likely only continue to increase as coronary CTA is now considered a reasonable option for screening for atherosclerosis in asymptomatic masters athletes [7]. The proposed recommendations in this paper deviate from the current ACC/AHA guidelines primarily in that it recommends all asymptomatic athletes in whom a bridge is discovered get stress testing, and those with high risk bridge characteristics undergo rhythm monitoring and yearly stress testing. Although the argument can be made that there is no evidence that obtaining these tests decreases the risk for sudden cardiac death, this is true for the testing obtained in most cardiovascular conditions in which further cardiac testing is obtained prior to an athlete returning to play. Additionally, exercise stress testing and heart rhythm monitoring typically take up less resources from a personnel and financial standpoint as opposed to more advanced testing like a cardiac MRI, which is needed for monitoring of other cardiovascular conditions in athletes.

## 8. Conclusions

Myocardial bridging is a congenital coronary variant whose clinical significance in athletes is substantially underappreciated and inadequately studied. The condition can cause exercise-induced ischemia, ventricular arrhythmia, and sudden cardiac death through hemodynamic mechanisms that are uniquely relevant to the physiological demands of athletic competition. Current pre-participation screening programs cannot detect MB, and even when MB is identified, no validated risk stratification framework exists to guide management in athletes.

Pharmacological management with beta-blockers or calcium channel blockers provides symptomatic relief but imposes exercise limitations in competitive athletes. Surgical myotomy offers a potentially definitive solution for refractory symptomatic patients, but procedural evidence derives from small retrospective series. Aggressive hydration is a conservative therapy that can provide symptomatic improvement based on limited case reports. Physical activity guidance—the most clinically pressing question—is currently answered by expert opinion in the near-total absence of prospective outcome data.

Athletes with MB and their physicians must currently make decisions under genuine uncertainty, a situation that is ethically and practically challenging. Transparent shared decision-making, individualized risk assessment, and regular reassessment represent the best available approach until robust prospective data emerge. The sports cardiology community has an urgent obligation to prioritize MB research, develop international collaborative registries, standardize diagnostic and management protocols, and generate the evidence that athletes, physicians, and governing bodies need to make informed decisions about participation in competitive sport.

## 9. Future Directions

Addressing the evidence gaps identified in this review will require a coordinated, international research effort across several domains:

Prospective Cohort Studies: Large-scale prospective studies of athletes with confirmed MB, stratified by hemodynamic significance and participation level are needed to define natural history, adverse event rates, and predictors of poor outcomes. Collaborative registries, analogous to those established for hypertrophic cardiomyopathy or arrhythmogenic cardiomyopathy in athletes, should be developed under the auspices of major sports cardiology organizations.

Standardized Diagnostic Protocols: International consensus on the optimal diagnostic pathway for athletes with suspected or confirmed MB—encompassing modality selection, functional assessment thresholds, and reporting standards—is needed to enable data pooling and comparison across centers. The role of emerging modalities, including dynamic CCTA, 4D flow MRI, and AI-assisted coronary imaging interpretation, should be evaluated in prospective studies.

Risk Stratification Tool Development: A validated, multivariable risk score incorporating anatomical, hemodynamic, clinical, and genetic parameters should be a priority research objective. Machine learning approaches applied to registry data may accelerate this development, provided sufficient sample sizes are achieved.

Therapeutic Trials: Even pragmatic, registry-based comparative effectiveness studies comparing medical therapy, myotomy, and conservative management in symptomatic MB—while not randomized—would represent a major advance over the current case series evidence base. Randomized trial design, though methodologically challenging, should be explored in collaboration with regulatory committees.

Wearable Technology Integration: The integration of ambulatory monitoring platforms—capable of continuous ECG, heart rate variability, and potentially photoplethysmography-derived surrogates of ischemia—into follow-up protocols for athletes with MB could provide real-world physiological data during training and competition that laboratory testing cannot capture. These data streams, combined with GPS-derived exercise intensity metrics, could transform our understanding of the relationship between exercise intensity and MB-related risk.

Guideline Development: The ESC, AHA/ACC, and national sports medicine bodies should prioritize the development of stronger and more specific evidence-based, MB-specific guidance for athletes—acknowledging current limitations while providing a clear framework that can be updated as evidence accrues. The current absence of phenotype-specific MB guidance leaves clinicians and athletes without the structured support they require.

## Data Availability

All data referenced in this document is publicly available and can be assessed through the papers references.
